# Evaluation of Tobacco Control Measures in the Organization for Economic Co-operation and Development Countries: A Comparative Study Using Data Envelopment Analysis

**DOI:** 10.34172/jrhs.2021.62

**Published:** 2021-09-05

**Authors:** Majid Safaei Lari, Behzad Raei, Pedram Nourizadeh Tehrani, Amirhossein Takian

**Affiliations:** ^1^Department of Health Management, Policy, and Economics, School of Public Health, Tehran University of Medical Sciences, Tehran, Iran; ^2^Department of Global Health and Public Policy, School of Public Health, Tehran University of Medical Sciences, Tehran, Iran; ^3^Health Equity Research Center, Tehran University of Medical Sciences, Tehran, Iran

**Keywords:** Efficiency, OECD, Taxes, Tobacco use

## Abstract

**Background:** This study aimed to measure the efficiency and productivity of tobacco control policies across 16 selected Organization for Economic Co-operation and Development (OECD) countries from 2008 to 2014.

**Study design:** A panel-data study.

**Methods:** Data envelopment analysis was used in this study. Taxation on tobacco products and pictorial warning labels were chosen as the inputs. Percentage of the population of daily smokers above 15 years old and the number of cigarettes used per smoker per day were output variables. Additionally, the Malmquist total factor productivity (TFP) was used to analyze the panel data and measure productivity change and technical efficiency changes over time.

**Results:** The highest technical efficiency score (1.05) was attributed to Norway, while the lowest (0.91) belonged to the UK. Technological change with a total mean of 1.06 implied that the technology and creativity have increased, while countries have been able to promote their creativity over the studied period. Norway with the TFP score of 1.15 was the most productive country, while the UK and Turkey with TFP scores of 0.95 and 0.98, respectively, were the least productive countries in terms of the implementation of the tobacco control policies.

**Conclusions:** Most OECD countries have productively implemented tax and pictorial warning policies to reduce tobacco use. To achieve the optimum outcome of the tobacco control policies and overcome the challenges of smoking use, countries need to tackle the difficult underlying factors, i.e. tobacco industry opposition and lobbyists, smuggling, and low socioeconomic status.

## Introduction

With a broad spectrum of adverse health effects, such as obstructive pulmonary disease and ischemic heart disease, tobacco use is a serious threat to global public health^1^ and a heavy economic and health burden on societies. As an estimate, the deaths of 5,000,000 adults globally were directly attributed to smoking in 2012, which is predicted to increase to 8,000,000 by 2030 ^2,3^.

Despite noticeable smoking rate reduction among the Organization for Economic Co-operation and Development (OECD) countries, it still remains the widest preventable risk factor for health^4^. In 1996, the World Health Organization (WHO) voted to execute the WHO Framework Convention on Tobacco Control (WHO FCTC) which was adopted in 2003 and finally came into force in 2005 ^5-7^. The so-called MPOWER introduced by WHO in 2008 included some preventive and control policies aimed at curbing tobacco use. MPOWER has six components: monitoring tobacco use and prevention policies; protecting people from tobacco smoke; offering help to quit tobacco use; warning about the dangers of tobacco; enforcing bans on tobacco advertising, promotion, and sponsorship; and finally raising tobacco taxes ^8^. Substantial tobacco tax and pictorial warnings are considered the most cost-effective interventions to reduce tobacco consumption ^8,9^.

High-income countries (HICs), i.e. OECD countries, usually impose higher taxes on tobacco products, such as cigarettes, compared to low- and middle-income countries (LMICs)^10^. Partially due to tax increases, cigarette consumption has become less affordable in HICs over time^11^. Pictorial warnings on tobacco products are also an effective way to promote consumer knowledge about the risks of tobacco use ^12^. Many smokers from several countries have been reported to get more awareness about the lethal and adverse risks of smoking from pictorial warning labels than other sources, except for television^9,13^. Furthermore, secondhand smokers, especially children, have been reported to be highly aware of warning labels^13^. A survey illustrated that smokers who noticed the warnings on cigarette packages were significantly more likely to confirm health risks of tobacco, including lung cancer and heart disease^14^.

However, globally, there are always some setbacks that obstruct the implementation of tobacco control policies. For example, the tobacco industry exploits a wide range of tactics to hamper governments from implementing tobacco control policies. The strong opposition from the tobacco industry undermines governments to prevent increasing taxes and pictorial warnings on tobacco products^15^. Moreover, governments might be impotent to adopt these policies on a large scale. Hereupon, it is important to ensure that these preventive policies are being implemented effectively and at any adoption levels, even limited levels, these policies result in the utmost reduction in tobacco use.

In this regard, the present study aimed to measure the total factor productivity (TFP) of tobacco control policies across 16 selected OECD countries during 2008-2014. The TFP is a result of technical efficiency (TE) multiplied by technological change (TCH). The TE changes show how efficient tobacco control policies have been implemented over this period. Moreover, the TCH shows the changes in technology and creativity in the implementation of the policies during these years. Data Envelopment Analysis (DEA) method was used to determine the performances of the countries^16^.

To the best of our knowledge, this is the first application of the DEA to measure the efficiency and productivity of preventive medicine policies and compare the efficiency of tobacco control interventions in a cross-country context. The cross-country comparisons can provide, we envisage, a useful and practical source of evidence for policy-makers to improve their performance in terms of making palatable policies^17^.

## Methods

###  Data envelopment analysis and Malmquist approach 

The DEA is a non-parametric method to measure relative efficiency^18^, which has been frequently used for measuring health system performance^19^. As a data-oriented approach, DEA can examine the performance of a set of decision-making units (DMUs) that transform multiple inputs into multiple outputs^20^. The DEA employs linear programming (LP) methods to calculate the efficiency measures relative to non-parametric frontiers^16^.

There are two versions of DEA: input-oriented and output-oriented. If the aim is to minimize available inputs to provide given levels of outputs, the model would be called input-oriented. On the other hand, if it is assumed that outputs are manageable and the target is to maximize outputs from given levels of inputs, then the model is called output-oriented^16^. Pictorial health warnings and taxes on cigarettes have been mentioned in the past as the most effective policies to control tobacco use^21,22^.

Hence, to promote efficiency, lowering inputs was found out to be an irrational decision. On the other hand, countries can concentrate on the outputs and improve them from the given input levels by engaging the other tobacco preventive policies (which were quoted in the introduction). Now it can be concluded with regard to definitions of the DEA orientations that an output-oriented version seems to be appropriate for this study^16^ as is formulated below^18^:


$$Maximize\;h_{o}=\sum_{r=1}^{s}u_{r}y_{rjo}+u_{o}$$

Subject to:


$$\sum_{i=1}^{m}v_{i}x_{ijo}=1$$

$$\sum_{r=1}^{s}u_{r}y_{rj}-\sum v_{i}x_{ij}+u_{o}\leq 0,\;j=1,...,n$$

$$u_{r},v_{i}\geq 0$$

$$u_{o}{\;}_{>}^{\leq }0$$

Here, yrjyrj is the amount of output r from DMU *j*, xij is the amount of input i to DMU *j*, ur is the weight given to output *r*, vi is the weight given to input *i,* n is the number of DMUs, s is the number of outputs, and m is the numbers of inputs.

 The sign of u0 shows the reveals returns to scale. In fact, DEA is based on two different models: variable returns to scale (VRS or BCC) or constant returns to scale (CRS or CCR). It should be noted that under the BCC models, returns to scales can change. If the proportions of increases in both inputs and outputs are the same, the return to scale is constant (u0=0). If outputs increase by a larger proportion than each of inputs, the returns to scale would be increasing (u0>0). Finally, decreasing returns to scale happen when outputs are larger than inputs by a smaller proportion (u0<0). Under the CCR model, returns to scale are always constant and do not change.

The DEA model is appropriate for a specific period, not overtime. It should be noted that the present study covers 2008 until 2014, and the efficiency, innovation or creativity, and technology in applying tobacco preventive policies might have changed during this period. Therefore, a DEA analysis of panel data was used across selected countries during the above-mentioned period. Consequently, TFP was measured using DEA-based Malmquist indexes framework^23^ which considered changes in both TE and technology of the tobacco control policies over time. In addition, a two-input, two-output model was also used. The Malmquist productivity index (MPI) structure is as below:


$$MPI={\left[\frac{OE/OG}{OC/OB}*\frac{OF/OG}{OA/OB}\right]}^{0.5}$$

**Figure 1:**
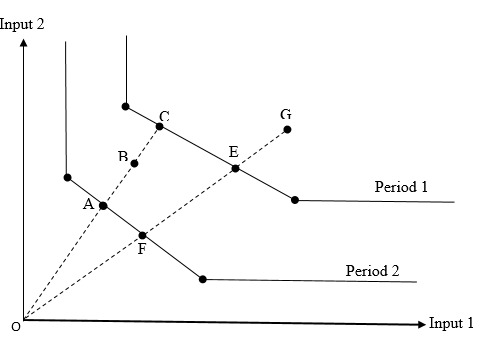
Malmquist Index

The MPI is calculated by the geometric mean of two different parts. The first expresses that the distance between the two production points, G and B (showing a country in the two periods), is measured relative to the production frontier of the first period (Figure 1). Based on the second factor, this time the distance of these production points (G and B) is measured relative to the production frontier of the second period. The score of the MPI is interpreted according to the following:

1. If the score was greater than unity (MPI>1), it would indicate the country has raised the productivity in implementing tobacco control policies.
2. If the score was equal to unity (MPI=1), then it would suggest the productivity is constant.
3. If the score was less than unity (MPI<1), therefore it would imply that country was less efficient in the second period, compared to the first period in terms of implementation of tobacco control policies.

 The MPI can be decomposed into two factors: TCH and change in TE (“catch-up”). Hence, according to this decomposition, the MPI will turn into:


$$MPI=\frac{OE/OG}{OA/OB}{\left[\frac{OA}{OC}*\frac{OF}{OE}\right]}^{0.5}$$

The first factor which is outside the brackets shows the TE of implementing tobacco control policies in both periods and measures the efficiency change while transferring from the first to the second period. It shows that the country will be more efficient (with a score greater than unity) provided it nears its production frontier; and conversely, if the country recedes from its production frontier, it will be less efficient and have a lower efficiency score (with a score less than unity). Neutrally, if the country stays in the same position relative to its frontier and does not move, the efficiency will be constant (with a score equal to the unit).

 The second factor in this MPI (inside the brackets) calculates transfers of the actual frontier between the two periods. A shift in the frontier means a change in technology and creativity of each country in terms of implementing tobacco control policies which depends, in turn, on the function of that country. The result of each function can be an increase in technology (frontier) with a score greater than the unit, a decrease with a score less than the unit, or maintenance of the same position with a score equal to the unit.

###  Variables and assumptions

Out of the six MPOWER policies, only two had numerical datasets and were included as the inputs in the model^9^. These two policies were the taxation of tobacco products and placement of pictorial warning labels on tobacco products. The others had mostly been expressed as “Yes” or “No”, meaning whether they had been executed or not, and statistical analysis was not conducted on them. The taxes on the most sold brand of cigarettes (taxes as a percent of price) were considered as measures of tobacco taxation. Pictorial warnings are percentages of principal display area mandated to be covered by health warnings (front and back of cigarette packaging). Variables of the outputs included the prevalence of smokers, with the measure of the population of smokers as a percentage of the population older than 15 years old who are daily smokers, and also the number of cigarettes used per smoker per day. To preserve the positive concept of outputs in the DEA models and also since the efficiency measurement techniques basically suppose that “more outputs are better” ^24^, the prevalence of smokers and the number of used cigarettes per smoker were conversely entered into the model

$$\left(\frac{1}{outputs}\right)$$.

The Malmquist indexes were calculated under both CRS and VRS; hence, there is no difference which one is selected^23^. Nonetheless, when the study design is cross-national and variables are expressed as ratios, the BCC model is preferable^25^. Therefore, in this study, the BCC model was selected. The countries and the period of panel data were selected based on the maximum data availability. Eventually, 16 OECD countries and four time points (2008, 2010, 2012, and 2014) were chosen. The OECD is an international organization with 38 members of high-income countries that works to build better policies for better lives.

According to previous studies, the efficiency depends on the number of degrees of freedom, meaning that if the number of **DMUs** (n) is less than the sum of inputs and outputs (m+s), most of the DMUs will be likely to be determined as efficient. They introduce a rough rule of thumb in the envelopment model which suggests the number of DMUs (n) should be equal to or greater than the max {m*s, 3*(m+s)}^16^. To observe this assumption in the present study, based on the rule of thumb, the number of DMUs is equal to 12 {12=3*(2+2)} which is less than 16 (the number of countries). The DEA-SOLVER-LV8 (2014-12-05) application was used for panel data analysis.

###  Data

The data were collected from WHO regarding the two input variables, panel data of pictorial warnings, and taxes on cigarette for all 16 countries during the four selected periods^26^. No data was found for pictorial warnings for the year 2008; hence, and the data from the year 2007 were used instead. Data about the prevalence of smokers and cigarettes used per smoker as output variables were collected from the OECD Health Database^27^. It should be mentioned that there were a few missing data points that were properly fixed using a single imputation method.

## Results

Table 1 summarizes the descriptive statistics of all four variables, including inputs and outputs. The mean value of areas covered by pictorial warnings on cigarette packaging had an increase of just 0.35% from 2008 to 2010, while it remained constant from 2012 to 2014. The main increase, which was 2.66%, occurred during 2010-2012. The mean of taxes on cigarette increased by 2.19% during the first period and afterward, experienced slight fluctuations during the other periods. The mean prevalence of smokers and cigarettes used per smoker also decreased during 2008-2014.

**Table 1:** Summary of descriptive statistics of variables.

**Variables**	**Mean**	**SD**
Pictorial health warnings		
2008	38.49	8.93
2010	38.84	8.40
2012	41.50	13.51
2014	41.50	13.51
Taxes on cigarettes		
2008	70.61	10.77
2010	72.80	9.96
2012	72.57	9.95
2014	72.89	10.09
Prevalence of smokers		
2008	22.29	3.90
2010	21.21	3.84
2012	19.95	3.50
2014	18.97	4.12
Cigarettes per smoker per day		
2008	14.05	2.38
2010	13.43	1.87
2012	13.31	2.99
2014	12.81	2.58

Results of the TE change or catch-up, TCH, and MPI for each country are tabulated in tables 2,3, and 4 respectively. The catch-up shows how far each country has transferred from the efficient frontier during the studied period. In fact, it means how efficiently taxes and pictorial health warnings on tobacco products have been implemented over this period. The total catch-up mean value was 0.98 which indicates that the transfers from the frontier have not been considerable and these selected OECD countries have not been successful in TE improvement. Hence, it can be said that the TE has slightly decreased overall. The highest (0.91) and lowest (1.05) TE scores were attributed to Norway and the UK. According to Table 2, all countries, except Denmark and the Czech Republic, had TE scores greater than the unity during the first period (2008-2010), while most of their scores declined during the next two periods. The United States and Denmark showed constant scores that were equal to unity over the three periods. The total mean of standard deviation was just 0.03, which seems to be very narrow.

**Table 2:** Summary of technical efficiency changes (catch-up).

**Catch-up**	**2008→2010**	**2010→2012**	**2012→2014**	**Average**
Canada	1.05	0.95	1.02	1.01
Denmark	1.00	1.00	1.00	1.00
Czech Republic	0.99	0.89	0.93	0.94
Estonia	1.03	0.88	0.99	0.97
Finland	1.16	0.98	0.93	1.02
France	1.08	0.88	0.94	0.97
Italy	1.07	0.91	0.94	0.97
Japan	1.00	0.99	0.99	0.99
South Korea	1.00	1.00	0.99	0.99
Latvia	1.02	1.24	0.67	0.97
Netherlands	1.13	1.00	0.80	0.98
New Zealand	1.19	0.95	0.93	1.03
Norway	1.16	1.00	1.00	1.05
Turkey	1.18	0.82	0.83	0.94
United Kingdom	0.94	0.91	0.90	0.91
United States	1.00	1.00	1.00	1.00
Average	1.06	0.96	0.93	0.98
Max	1.19	1.24	1.02	1.05
Min	0.94	0.82	0.67	0.91
SD	0.08	0.09	0.09	0.03

**Table 3:** Summary of technological changes

**Frontier**	**2008→2010**	**2010→2012**	**2012→2014**	**Average**
Canada	0.99	1.05	1.10	1.05
Denmark	0.92	1.35	1.01	1.09
Czech Republic	0.92	1.12	1.15	1.06
Estonia	0.96	1.13	1.15	1.08
Finland	0.91	1.11	1.16	1.06
France	0.94	1.12	1.15	1.07
Italy	0.94	1.12	1.16	1.07
Japan	1.11	0.93	1.05	1.03
South Korea	1.08	1.05	1.03a^a^	1.05
Latvia	0.98	1.15	1.16	1.10
Netherlands	0.97	1.11	1.17	1.08
New Zealand	0.98	1.07	1.10	1.05
Norway	0.96	1.12	1.20	1.10
Turkey	0.96	1.10	1.10	1.05
United Kingdom	0.85	1.12	1.16	1.04
United States	1.06a^a^	1.02a^a^	1.03a^a^	1.04
Average	0.97	1.10	1.12	1.06
Max	1.11	1.35	1.22	1.10
Min	0.85	0.93	1.01	1.03
SD	0.06	0.084	0.06	0.02

^a^ indicates that an infeasible LP problem occurred for computing this number.

**Table 4:** Summary of Malmquist index

**Malmquist**	**2008→2010**	**2010→2012**	**2012→2014**	**Average**
Canada	1.05	1.01	1.12	1.06
Denmark	0.92	1.35	1.01	1.09
Czech Republic	0.91	1.01	1.08	1.00
Estonia	1.00	1.01	1.14	1.05
Finland	1.06	1.09	1.09	1.08
France	1.01	0.99	1.09	1.03
Italy	1.00	1.03	1.10	1.04
Japan	1.11	0.93	1.05	1.03
South Korea	1.08	1.05	1.03a^a^	1.06
Latvia	1.00	1.43	0.78	1.07
Netherlands	1.10	1.11	0.93	1.05
New Zealand	1.18	1.02	1.03	1.08
Norway	1.12	1.12	1.22	1.15
Turkey	1.13	0.90	0.92	0.98
United Kingdom	0.80a^a^	1.02a^a^	1.04a^a^	0.95
United States	1.06	1.02	1.03	1.04
Average	1.03	1.07	1.04	1.05
Max	1.18	1.43	1.22	1.15
Min	0.80	0.90	0.78	0.95
SD	0.09	0.13	0.10	0.04

^a^ indicates that an infeasible LP problem occurred for computing this number.

 Conversely, the total mean of TCH was 1.069 which implies that the technology and creativity in the implementation of taxes and pictorial health warnings on tobacco products have risen over the studied period. This means that the selected countries have been able to promote their creativity. Norway and Japan had the maximum (1.105) and minimum (1.03) mean values in terms of technological change, respectively. During the first period, all countries, except Japan, South Korea, and the United States, showed TCH scores less than unity. During the second period, the United States and South Korea stayed above unity, while other countries improved their TCH scores up to greater than unity, except for Japan whose technological score fell below unity. Eventually, during the third period, all countries showed TCH scores greater than one. The total mean standard deviation for TCH was 0.02, which was not considerably high.

 Finally, the MPI value of 1.05 could be recognized as a great deal between TH changes and TCHs. The MPI or that TPF achievement is a deduction of multiplied TE by TCH. Since this value was greater than unity, it means that there was an overall productivity growth. Only the UK and Turkey experienced a decrease in their productivity. The maximum TFP with the value of 1.15 was related to Norway, which presented the best performance in terms of engaging inputs and producing outputs. The standard deviation of MPI was calculated at 0.04, which was not significant.

## Discussion

 The endorsed MPOWER of WHO renders effective policy interventions to restrict tobacco consumption. The FCTC parties are committed to implementing most of these measures to various extents. This study measured the TE change, the TCHs, and consequent changes in the productivity of two MPOWER policies, namely tobacco tax raising and pictorial warning, in selected OECD countries during four time slots from 2008 to 2014.

The annual mean Malmquist index of included countries, except for Turkey and the UK, from 2008 to 2014 was more than one, that is, the TFP showed an upward trend. Among them, the main reason for the decline of TFP in Turkey and the UK was the regression of TE, implying the necessity of enhancing TE by optimizing resource allocation and improving the management. The results indicated that the main reason for the reduction of TFP was inefficiency. However, it should be noted that Turkey gradually increased the tax on tobacco products during 2005-2011; accordingly, the tax rate rose from 58% of the retail price to 65%, and the price of cigarettes was more than tripled. In addition to the pricing policy, Turkey followed non-pricing policies, all of which led to a >4% drop in tobacco prevalence during 2008-2012^28^. Additionally, between 2012 and 2014 Turkey switched from ad valorem to mixed system taxation, which helped converge prices across different types of a given tobacco product^29^.

In the present analysis, despite the fact that the UK had a Malmquist score of less than 1, a survey indicated that a high percentage of smokers in this country are supporting tobacco control measures, such as taxation ^30^. Nevertheless, tobacco companies in the UK have formulated a plan for cross-subsidizing less expensive brands to capture market shares^31^. Moreover, there is evidence suggesting that tobacco companies in this country have lobbied politicians to change legislation in favor of their business^32^.

Regarding the effectiveness of tobacco control policy, Joossens and Raw in 2005 ranked 30 European countries based on their total score on the 100-point scale. They found that only four countries, including Iceland, Ireland, Norway, and the UK, scored 70 or more^33^. The authors iterated their study in 2010 and noticed that the aforementioned countries attained the highest rank order again and that Turkey was one of the countries that were doing well with a score of 56^34^, which somewhat agrees with the findings of the present study.

Despite the fact that the UK is among the countries where taxes represent around 80% of the retail price, there is a huge gap between the final prices of various tobacco products^35^.^.^Some smokers tend to use low-priced brands (brand switching) in response to the increased price of tobacco products^36^. Currently, in all countries, taxation on roll-your-own cigarettes is lower than manufactured ones. This discrepancy has exacerbated brand switching, especially in countries, like the UK, where 32% of smokers are smoking hand-rolling tobacco. Illicit tobacco trade and smuggling are other problems that the UK is facing. All of these issues lead to the weaker effectiveness of tobacco control policies. Findings of the present study revealed that Norway has experienced the most productive tobacco control directives, compared to other included countries. It is worth noting that this country has the highest cigarette prices in the world with a negligible illicit trade (only 1% smuggling)^31^.

Most countries that acquired an increase in the catch-up effect during 2008-10, experienced a decrease in that effect over the next years. Taxation might have a three-fold effect on tobacco use, including a barrier to the initiation, reduction of consumption among current smokers, and preclusion of former smokers from relapsing^37^. Detailed data on different dimensions of tax policy, including tax administration and tax structure, can inform researchers and strategists to advance related tax policies around the world^11^. In addition, the effectiveness of implementing a strategy is not guaranteed and may vary depending on fluctuating circumstances. For example, in countries where access to low price, untaxed, and inexpensive tobacco products is high, low-income tobacco users show less sensitivity to price changes. However, populations with a higher proportion of younger smokers, especially new starters, might be more sensitive to tax and price policies than adult smokers^21^.

The effect of increased tobacco prices on smoking prevalence varies depending on the characteristics and interests of the population within various settings. Heterogeneity in price responsiveness might be explained by factors, such as the level of addiction of smokers, cigarette affordability, tobacco industry activity to encourage consumers and product substitution due to availability of a great variety of tobacco products and wide price^38^. Various factors, including tobacco industry price discounting strategies and proactive lobbying and price-reducing marketing in the OECD countries, may explain the variance in the effectiveness of MPOWER interventions^39,40^. Furthermore, the existence of state-owned tobacco companies implies a complex and ambiguous attitude towards smoking. As long as governments continue to generate significant revenue from monopoly tobacco production, they will face serious inconsistencies in how they deal with the adverse health consequences of tobacco use, e.g. the prevalence of tobacco-related illnesses and mortality^41^. This might in turn indicate the need to take strong actions regarding the adaptation of a range of tactics for appropriate implementation of FCTC by WHO^42^.

According to the WHO estimates, higher taxes, depending on their types, can contribute to almost half of the reduction in smoking. For instance, ad valorem taxes are built upon prices; hence, tobacco companies can potentially undermine the effects of higher taxes by reducing supply and putting lower prices on tobacco products. Therefore, industry pricing strategies could manipulate consumption levels and change tax revenue. Alternatively, specific excise taxes imposed based on the quantity of products to generate a fixed tax amount must match or outpace periodical inflation to meet their tobacco control objectives^29^. Hence, many aspects of each instrument included in the MPOWER package are essential to consider when assessing the merits of designated tools^11^.

Findings of this study revealed that all included countries have been following an upward trend towards TCHs, which led to positive performance during 2012-14. Such progress reflects innovations and the use of new technologies in the implementation of pictorial warning policies. The greatest TCH belonged to Norway, while Japan showed the lowest change. The main elements, including the feature of graphic design on cigarette packs, the size of the space covered by health warnings, and the periods for label rotation, may account for the impact of pictorial health warnings on smoking prevalence^11^. Nevertheless, problems, such as the sale of single sticks of cigarettes, could reduce the effectiveness of health warnings on the packs.

Investigation in technology plays a key role in combating the illicit trade of tobacco products. Many customs agencies have realized the need to employ technology tools and sniffing dogs. The emergence of low-cost technologies, such as mobile phones, and system-level interventions, including e-health file technology in identifying tobacco users, the timely intervention of clinicians, and directing interventions to evidence-based treatment algorithms, can successfully facilitate smoking cessation interventions worldwide^11^.

 Another key finding from this study was that the Malmquist index for most countries progressed in the TFP over the study period. Most of the countries with a Malmquist index above experienced an increasing trend in innovation and technology use. Nevertheless, the observed differences in the progress of tobacco control activities among the selected countries might be related to the comprehensiveness of the MPOWER package, which might have, in turn, led to the various extents that a particular country has pursued the FCTC goals.

Norway implemented the point-of-sale tobacco display ban in 2010. This may be the impact of the increase in the use of TCHs in this country during 2010-2012, compared to the previous period. Consumers declared that the ban prevented young people from beginning smoking and also helped cessation endeavors^43^. Norway, as the vanguard in this study, has also applied the strongest levels of monitoring, mass media or anti-tobacco campaigns, and smoke-free policies.

The path from policy to reduction in tobacco consumption depends on the possibility of implementation of tobacco control measures in a country, and also on the effectiveness of those measures. Despite the progress observed in recent years, no government is fully implementing the MPOWER strategy. Many challenges have remained and much more needs to be done to stop one of the worst scourges of modern times. Application of restrictions on all forms of tobacco advertisement, promotion, and sponsorship are among the most effective solutions that few countries have adopted successfully^44^.

###  Limitations and strengths

The strength of this study was being the first in its kind to measure the efficiency and productivity of MPOWER policies in the OECD countries using robust methods. However, it should be noted this assessment was limited. This study provided some insight into the issues associated with tobacco control measures for decision-makers to translate good policy models into tangible action and results. Comparative productivity is an effective methodology as well as an indicator to elucidate the existing circumstances in any country. Nevertheless, the interpretation of such comparisons for a more comprehensive status requires careful attention to other dimensions. Tobacco industry opposition and lobbyists, smuggling, financial barriers, like the economic benefit of tobacco production, and the high cost of cessation programs, might have overshadowed the successful tobacco control plans^3^. Socio-economic situations, poverty, and low education levels are also major hindrances to access cessation interventions and acquisition of knowledge about the harmful effects of smoking^45,46^.

## Conclusions

 Most OECD countries have productively implemented MPOWER policies to reduce tobacco use. Based on the results, MPOWER interventions were not the sole reason for the dissatisfying productivity results. To achieve the optimum outcome of the FCTC MPOWER policies and overcome the challenges of smoking use, countries need to tackle the difficult underlying factors, i.e. tobacco industry opposition and lobbyists, smuggling, and low socioeconomic status, which may hinder the meaningful implementation of such policies and eventually undermine sustainable development goals.

## Acknowledgments

 None.

## Conflict of interests

 The authors have no conflicts of interest associated with the material presented in this paper.

## Funding

 None.

## Highlights

- Most Organization for Economic Co-operation and Development countries have productively implemented MPOWER policies to reduce tobacco use.
- Technology, innovation, and creativity have played more important roles than efficiency in productive countries.
- Productive and meaningful tackling of the tobacco use problem also depends on opposition with the tobacco industry, lobbyists, smuggling, and low socioeconomic status.
